# Development of a survey form through Delphi study about adverse events associated with the miniscalpel needle, for application in prospective observational studies regarding safety of miniscalpel needles: Study protocol

**DOI:** 10.1097/MD.0000000000012736

**Published:** 2018-10-12

**Authors:** Sang-Hoon Yoon, Haebeom Lee, Chan-Young Kwon, Damin Jeon, Hyunho Kim, Hee-Geun Jo, Aesook Shin, Younghee Yun, Jae-Uk Sul, Geon-Mok Lee, Jun-Hwan Lee, Jungtae Leem

**Affiliations:** a Chung-Yeon Medical Institute; b Chung-Yeon Korean Medicine Hospital, Gwangju; c Department of Human Informatics of Korean Medicine, Interdisciplinary Programs, Kyung Hee University; d Inae Korean Medicine Clinic, Seongdong-gu; e Department of Clinical Korean Medicine, Graduate School, Kyung Hee University, Dongdaemun-gu; f Dongshin Korean Medicine Hospital, Yangcheon-gu; g Research and Development Institute, CY Pharma Co, Gangnam-gu; h Lee-Geonmok Wonli Korean Medicine Hospital, Seocho-gu, Seoul; i Clinical Medicine Division, Korea Institute of Oriental Medicine; j Korean Medicine Life Science, University of Science and Technology (UST), Campus of Korea Institute of Oriental Medicine, Daejeon, South Korea.

**Keywords:** acupotomy, adverse event, Delphi technique, miniscalpel needle, patient safety, safety management

## Abstract

**Background:**

Despite the wide usage of miniscalpel-needles (MSNs), information about MSN treatment-related adverse events (AEs) is insufficient. As the definition of AE might vary among physicians, without an exact definition for pain and hemorrhage, it is difficult to provide accurate information about AEs in MSN treatment to physicians, researchers, and patients. The aim of our study is to reach a consensus about the items and definitions of AEs that should be included in the survey form for prospective observational multicenter studies to record MSN treatment-related AEs. We will especially focus on obtaining a consensus on the definition of pain and hemorrhage caused by MSN treatment.

**Methods:**

Our study protocol is composed of 6 steps. First, we will identify the aim of the study. Next, we will conduct a systematic review to investigate MSN treatment-related AEs reported till date in Korea. Third, we will conduct a pilot observational prospective single-center study on AEs in MSN treatment. We will develop a standardized case report form to record MSN treatment-related AEs, including the causality, severity, and details of the MSN procedure at every site. Next, based on the pilot study, the Delphi study questionnaire will be developed by a panel composed of 13 physicians. The Delphi study will have 4 rounds with open questions and 4-point Likert-scale closed questions. Through these rounds, we will develop a consensus about the items and definitions of AEs that should be included in the survey form for future multicenter studies about MSN treatment-related AEs. Following this, a face-to-face consensus meeting will be held for a final agreement of survey form. The final survey form will then be approved by the related academic society for dissemination.

**Discussion:**

The aim of this protocol is to develop a survey form for future prospective observational multicenter studies on MSN treatment-related AEs. This protocol will present the research methodology for developing a survey form, which will improve consistency and reliability between MSN treatment studies. We believe that this protocol can evaluate the safety of MSN treatment.

**Trial registration:**

Clinical Research Information Service: KCT0002849.

## Abstract 2

Supplemental Digital Content is available in the text

## 1 Background

**Figure 1:**
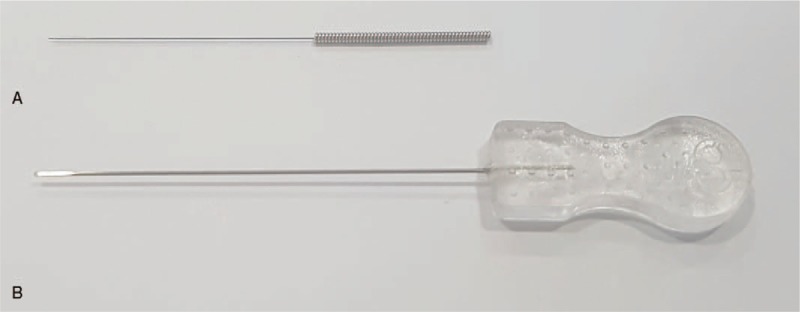
Structure of the miniscalpel needle. A, Filiform needle: 0.25 mm × 30 mm. B, Miniscalpel needle 0.5 mm × 50 mm.

The miniscalpel-needle (MSN) is a type of acupuncture device, with a flat knife attached to the tip of the needle.^[1]^ Currently, it is widely used for chronic musculoskeletal pain, and the therapeutic effect and treatment mechanism of MSN are being studied.^[2–5]^ MSN, however, might have a higher risk of adverse events (AEs) than traditional acupuncture, because the knives attached to the tip of the needle are thicker than the common traditional filiform needle (Fig. 1). Severe AEs, such as headache requiring hospitalization due to dural damage, were also reported after MSN treatment^[6]^; these are an infrequent occurrence with the common filiform needle. Till date, few incidents of AEs have been reported after MSN treatment^[7,8]^; however, no prospective observational study on MSN treatment-related AEs has been performed. On the other hand, occurrence of minor AEs has sometimes been omitted from study reports.^[4,9,10]^ Therefore, the exact incidence and severity of MSN treatment-related AEs is unknown.

The most common AEs reported after MSN treatment are needling pain, hemorrhage, and ecchymosis. Additionally, hemorrhage and needling pain are also the most common AEs reported in previous acupuncture safety studies.^[11,12]^ However, as acupuncture is a procedure involving needle insertion into the body, there is controversy as to whether mild pain and mild bleeding after treatment should be regarded as AEs.^[13,14]^ Hence, the reporting criteria for AEs in previous prospective studies have been inconsistent. One study has reported any incidence of bleeding to be an AE of acupuncture treatment.^[15]^ In contrast, some studies have considered only bleeding that lasted more than 10 seconds despite manual pressure to be an AE.^[16,17]^ Similarly, the criteria for reporting needling pain also varied among studies.^[15,17,18]^ The incidence of AEs after acupuncture treatment in prospective studies varied from 0.14% to 15%.^[12,18–21]^ The heterogeneity of reporting criteria for AEs may be a major reason for this. MSN treatment cannot avoid such controversy. The MSN is thicker than the traditional common filiform needle, and a 0.8 mm knife is attached to the tip of the needle; hence, it is often accompanied by pain and hemorrhage.^[22]^ If the reporting criteria for AEs are not clear, the same response may be regarded as an AE in some studies but not in others. However, there is no consensus on the reporting criteria for MSN treatment-related AEs. As a result, the incidence of AEs varied from 0% to 100% for each study.^[6,23–26]^

According to the International Conference on Harmonization of Technical Requirements for Registration of Pharmaceuticals for Human Use, the definition of an AE is *“*any untoward medical occurrence in a patient or clinical investigation subject administered a pharmaceutical product and it does not necessarily have a causal relationship with this treatment.*”*^[27]^ However, in case of procedures such as MSN treatment, which are almost always accompanied by pain and hemorrhage, we should set clinically significant reporting criteria for treatment-related AEs to provide meaningful information to clinicians, researchers, and patients. AEs should be reported only if the severity meets the pre-established criteria. To conduct prospective observational studies about AEs, it is necessary to reach a consensus about the reporting criteria for treatment-related AEs. Only after a consensus is established, clinically relevant information about safety can be provided. A checklist for AEs in conventional traditional acupuncture was published,^[28]^ but acupuncture and MSN treatments are different in terms of the anticipated AEs. The existing checklist does not define the reporting criteria for each AE.^[28]^

Reporting bias with regard to AEs can be reduced by applying pre-established criteria. In this study, a survey form for prospective observational studies on MSN treatment-related AEs will be developed through a Delphi study method. Consensus on the items and definitions of AEs in the survey form will be reached by expert discussion. Approval of the survey form from the relevant academic society will be acquired. The developed survey form will be used in future prospective, observational, multicenter, web-based studies to provide more accurate information on safety in MSN treatments.

## 2 Methods/design

### 2.1 Objectives

The aim of our study is to reach an agreement about the items and definitions of AEs that will be included in the survey form for prospective observational studies on MSN treatment-related AEs.

We will use the Delphi survey methods, especially focusing on what kinds of AEs should be included in the survey form, what kinds of AEs need to be defined by consensus, what kinds of criteria for defining AEs require consensus, and what are the final agreed definitions of the AEs.

**Figure 2:**
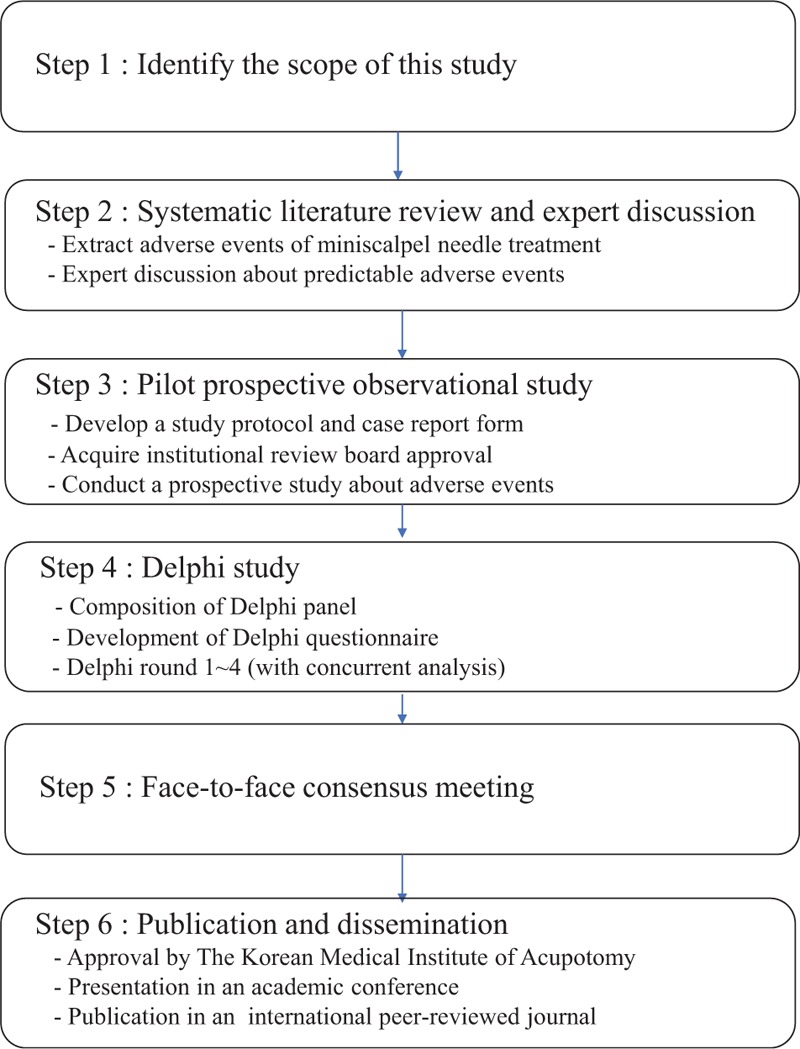
Study flow chart.

When we developed the Delphi study protocol in our research, we followed the Core Outcome Measures in Effectiveness Trials (COMET) handbook version 1.0.^[29]^ It consists of the following 6 steps: identify the scope of this study, a systematic literature review and expert discussion, pilot prospective observational study, a Delphi study, face-to-face consensus meeting, and publication and dissemination. The flow chart of our study is presented in Fig. 2.

## 3 Trial design and study setting

### 3.1 Phase I: Identify scope

The purpose of our study is to develop a survey form that will be used in prospective observational studies on MSN treatment-related AEs. The types and definitions of AEs to be included in the survey form will be decided based on expert consensus. A case report form (CRF) for prospective observational studies will be developed based on the survey form. The survey form will also be utilized in clinical practice. The scientific, technical, and ethical aspects of the research, including scope, were approved by the funding body (Korea Institute of Oriental Medicine).

### 3.2 Phase II: Systematic literature review and expert discussion

A systematic literature review will be conducted to investigate the incidence and types of MSN treatment-related AEs in Korea, and to assess their severity and causality. Electronic medical databases including Medline (via PubMed), EMBASE, Cochrane Central Register of Controlled Trials (CENTRAL), Oriental Medicine Advanced Searching Integrated System (OASIS), Korean Traditional Knowledge Portal (KTKP), KoreaMed, Korean Studies Information Service System (KISS), National Digital Science Library (NDSL), and Research Information Sharing Service (RISS) will be searched. In addition, to prevent omission of articles, manual searches will be performed on Google Scholar and the journal websites of 7 relevant societies in Korea, including Journal of Acupuncture Research, Journal of Pharmacopuncture, Korean Journal of Oriental Physiology & Pathology, Korean Journal of Acupuncture, Journal of Oriental Rehabilitation Medicine, Journal of Korean Medicine, and The Journal of Korea CHUNA Manual Medicine for Spine and Nerves. The searches will be conducted using “Dochim (Dochim) (in Korean),” “Chimdo (Chimdo) (in Korean),” “acupotomy, ”“miniscalpel,” and “miniscalpel” as search terms. All types of clinical studies which used MSN treatment as an intervention and were conducted in Korea will be included. Data on MSN treatment-related AEs, including its incidence and types as well as the descriptions of safety precautions and infection prevention measures, in each article will be extracted. Data for the MSN treatment procedure used will also be extracted using the Standards for Reporting Interventions in Clinical Trials of Acupuncture (STRICTA) guidelines.^[30]^ The STRICTA checklist will be helpful for structured data extraction. The quality of data regarding AEs will be assessed using the methodology from a previous study evaluating the safety of acupuncture.^[31]^ In addition, the severity and causality of AEs will be assessed using the Common Terminology Criteria for AEs (CTCAEs) scale^[32]^ and the World Health Organization-Uppsala Monitoring Centre (WHO-UMC) causality assessment system,^[33]^ respectively. The incidence of reported AEs will be quantitatively synthesized and presented as the incidence of AEs per 1000 individuals and 1000 treatment sessions with 95% confidence intervals (CIs). Two independent reviewers will perform the study selection, data extraction, and quality assessment of the data on AEs. Any disagreement will be resolved through discussion under the arbitration of the third author.

Using the systematic review results and previously developed checklist for AEs in acupuncture,^[28]^ a draft of the CRF for a pilot prospective study on MSN treatment-related AEs will be developed by expert discussion. AEs will be categorized as systemic and local. Predictable MSN treatment-related AEs will be discussed by experts; feasibility for pilot observational study will also be considered.

### 3.3 Phase III: Pilot prospective observational study

We will conduct a pilot prospective observational single-center study to explore the sample size, feasibility, and consideration for future original research. Based on the outcome of the pilot study, we will develop a protocol for original web-based multicenter prospective studies and develop standard operating procedures (SOPs). The pilot study was approved by the institutional review board (IRB) of Chung-Yeon Korean Medicine Hospital (IRB No. CYIRB 2017-12-003). The study was registered on Clinical Research Information Service (KCT0002666), which is one of the primary registries of International Clinical Trials Registry Platform (ICTRP). The study was designed according to Strengthening the Reporting of Observational Studies in Epidemiology (STROBE) statement.^[34]^ Inclusion criteria are as follows: age between 18 and 90 years and undergoing MSN treatment at the Chung-Yeon Korean Medicine Hospital after the research approval, ability to communicate with researchers with minimal help and ability to report adverse reactions, voluntary informed consent after explanation of the research, and complete demographic information on the medical records. The CRF for the study was developed based on the results of the systematic review (Phase II) and previously developed “Survey Form for Adverse Events Associated with Acupuncture and Moxibustion” by Kim et al.^[28]^ The CRF was finally modified by expert discussion. In the CRF, age, sex, diagnosis, and medical and surgical history of the patient will be recorded. To assess the relationship between MSN treatment procedure and possible AEs, insertion depth, location, stimulation, response of patients, and concomitant intervention at each treatment site will be recorded. Primary outcome will be the proportion of AEs after the MSN treatment. We will investigate the incidence of AEs twice. First, we will investigate AEs immediately after the completion of the MSN procedure followed by a telephonic investigation within 3 days after treatment. Two independent investigators (clinical research coordinator and the physician) will record the details of the AEs. The term AE will follow the Medical Dictionary for Regulatory Activities (MedDRA), which is an international standardized medical terminology dictionary for regulatory authorities and pharmaceutical companies.^[35]^ Severity of the AEs will be assessed by the 2 independent investigators according to the CTCAEs scale,^[32]^ and causality of the AEs will be assessed according to the WHO-UMC causality assessment system.^[33]^

Based on the outcome of the pilot observational study, SOPs for assessment and recording of MSN treatment-related AEs will be developed for future studies. The CRF will be modified based on the pilot study outcome, considering feasibility and reliability of the data collection. We will develop the first round of Delphi study questionnaire from the modified CRF, considering expert opinion.

### 3.4 Phase IV: Delphi study

The Delphi study was planned according to Guidance on Conducting and REporting DElphi Studies (CREDES).^[36]^ As the Delphi technique is a flexible method, it could be adjusted to the respective research purposes. Any modifications should be justified by a rationale and be applied systematically and rigorously

### 3.5 Preparing

The Delphi method is a set of procedures that derive reliable experts group consensus on the problem to be solved. It is commonly used in social science fields.^[37]^ We will follow the method of the previous Delphi study conducted by our research team.^[38]^ We acquired IRB approval exemption from the Chung-Yeon Korean Medicine Hospital IRB as Phase IV (Delphi survey) does not include any patients but only experts and clinicians in this field (CYIRB No 2018-07-001). In Delphi study, sample size calculation is not required but depends on the nature of the study. Previous literature recommend a panel of 10 to 18 experts.^[37]^ Therefore, we will recruit 13 members, who use MSN treatment in clinical practice and have experience of research according to the inclusion criteria, to the Delphi panel. Convenience and snowball sampling will be used. The criteria for inclusion to Delphi panels are as follows: licensed to practice as a Medical Doctor of Korean medicine after 6 years of study in the Korean Medicine college, clinical experience of more than 5 years, using MSN treatment in clinical practice more than 1 year, experience in research with authorship of at least 1 peer-reviewed article (including thesis), and voluntary consent to participate in the study. We will send the Delphi study questionnaire by e-mail to the 13 panel experts with a reply period of 2 weeks for each round. No research member has a conflict of interest.

### 3.6 Questionnaire

The Delphi questionnaire will comprise 4 rounds. We will use both open questions and closed questions. For closed questions, we will use the 4-point Likert scale in which 1 point means “strongly disagree,” 2 point means “somewhat disagree,” 3 point means “somewhat agree,” and 4 point means “strongly agree.” We will eliminate neutral response to force either a yes or no response to each question.^[38]^

In Delphi round 1, based on the CRF used in the pilot observational study, we will ask the panel experts questions like, “what kind of MSN treatment-related AEs should be included in the survey form for future MSN safety research studies” and “Do you think we require an additional consensus on the definition of the AEs” for each AE item via closed questions. In round 1, several open questions will also be used to encourage generation of ideas.^[39]^ In open questions, we will ask questions like, “What do you think should be the proper definition of pain and hemorrhage in safety research,” “What should be included in the definition criteria when defining pain and bleeding,” “In addition to hemorrhage and pain, what other AEs need a consensus for definition (Write with the proper definition).” The pilot version of the round 1 questionnaire about types and definitions are excerpted in Supplementary 1. In Delphi rounds 2, 3, and 4, we will continue the consensus process for the types and definitions of AEs in survey forms, except the AEs for which a consensus has been reached (agreement of more than 10 panel experts, explained later). The questions in round 2 will be generated based on the analysis of responses of the previous round. From round 2, an individualized questionnaire will be sent to each panel expert. In the individualized Delphi study questionnaire, we will display individual responses and the overall summarized response from the previous Delphi round. In closed questions, a minority commentator continues one's opinion; he or she will be asked to explain why the panel would like to continue one's comments. If required, open questions will be used in rounds 2 to 4. In the event of a lack of consensus regarding some items by round 4, it will be discussed in the face-to-face consensus meeting (Phase V). The response will be quasi-anonymous. The research teams will know the response, but the panel experts will not.

### 3.7 Analysis method

Content validity concerns “the degree to which a sample of items, taken together, constitute an adequate operational definition of a construct.”^[40]^ Content validity ratio (CVR) developed by Lawshe (1975) means “a linear transformation of a proportional level of agreement on how many “experts” within a panel rate an item “essential.” ^[41]^ CVR will be calculated according to Eq. (1).^[41]^
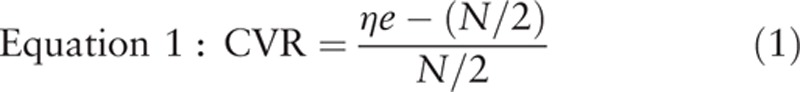


where N refers to the total number of panel experts (in our study, N = 13) and n_e_ refers to the number of panel experts who indicate that the item is essential (in our study, number of panel experts who select 3 point [somewhat agree] or 4 point [strongly agree]). A CVR value greater than zero indicates that more than 50% of the panel members have agreed that an item is essential. However, it is important to consider that more than 50% of panel agreement might occur by chance. Therefore, Lawshe developed CVR_critical_ (lowest level of CVR which indicates that agreement of experts exceeds that of chance for a given item with a type I alpha error suggested to be 0.05 with a one-tailed test).^[41]^ CVR_critical_ could be utilized to determine how many panel members need to agree to decide whether the item is essential or not. In our study, the number of panel experts is 13; CVR_critical_ is 0.538; and required minimum number of experts to agree that an item is essential for inclusion is 10, according to Ayre and Scally's table (2014).^[41]^ If 10 panel experts agree to the item, the proportion of agreement is 0.769 (10/13). It means that if 10 or more experts agree on the item in our study, we could be assured that the level of agreement is above chance. Thus, in our study, 10 is the critical number of panel experts required for agreement that an item is essential. As CVR_critical_ indicates level of agreement above chance, it is appropriate to test the hypothesis in one direction. Therefore, we did not adopt Wilson's Table which was based on a 2-tailed test.^[42]^

We will also use the degree of consensus (Equation 2) and degree of convergence (Equation 3) index as secondary indices for Delphi study. These could judge the extent to which the panel's consensus and convergence is reached.^[38]^ However, these indices will only be used as adjuncts.
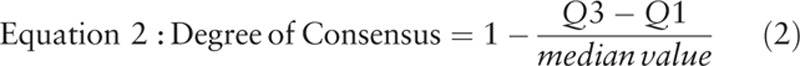

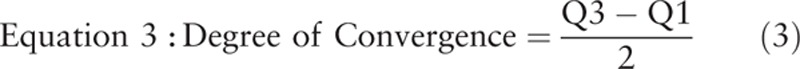


where Q1 and Q3 are the first quartile and the third quartile coefficients, respectively. In a previous study, when the degree of consensus was 0.75 or more and the degree of convergence was 0.5 or less, it was judged that agreement among experts was achieved. We will follow the criteria of above study.^[43]^

### 3.8 Phase V: Face-to-face consensus meeting

Prior to finalizing the survey form, a consensus meeting will be held to reach a final consensus on Delphi study results. Research team members and every Delphi study member will be invited to the face-to-face consensus meeting. The results of all prior Delphi rounds will be provided. In the Delphi survey, we will try to reach a consensus on the types and definitions of AEs that should be included in the survey form. However, we anticipate that there may be certain items on which a consensus is not reached. In the face-to-face consensus meeting, we will try to reach an agreement on the types and definitions of AEs by discussion. After sufficient discussion, we will conduct 1 round of closed Delphi survey with live polling software to ensure anonymity.^[44]^ We will follow the definition of consensus used in Phase III (Delphi survey). We will also discuss other parts of the case report form that will be used in our planning observational search (not the pilot). We will discuss in detail the process of (planning) the observational study, based on our pilot research experience and SOPs.

### 3.9 Phase VI: Publication and dissemination of results

After the consensus meeting, the developed survey form will be approved by The Korean Medical Institute of Acupotomy. It will be recommended to the members of the academy for use in their clinical practice and research. A final report of the Delphi study will be submitted to the funding body (Korea Institute of Oriental Medicine). The results of the study will also be published with the final survey form in the open access, peer-reviewed international journal. In the resultant article, we will recommend the use of the survey form in clinical practice and research. To disseminate our survey form, we will also present the results of our Delphi survey at an academic conference. We anticipate that with the assistance of researchers and physicians who are interested in MSN treatment, the survey form will be widely used. The final survey form will be used in the prospective observational study on the safety of MSN treatment. The protocol of the prospective study will be published and the survey form will also be provided as supplementary data. The survey form will be adopted in the case report form of the prospective study. Our survey form and results of the Delphi survey will be available from the research team upon request, after the results of our study have published. After an original AEs survey form is developed in Korean, the final form will be translated into English by a bilingual licensed Korean medicine doctor who has experience of more than 10 years in clinical practice and research.

## 4 Discussion

Currently, MSN treatment is being widely used for several diseases, but its safety has never been properly evaluated. There is no standard for reporting criteria of MSN treatment-related AEs. The absence of standardized reporting criteria may result in a reporting bias. This can affect the assessment of safety of the MSN treatment, making it impossible to properly define its risks and benefits.

Using the proposed survey form developed by Delphi study method will reduce the inconsistency in reporting MSN treatment-related AEs. It could be used as a standard form in clinical trials using MSN treatment. We also found that the definition and reporting criteria for AEs varied among earlier acupuncture safety studies. A systematic investigation of earlier acupuncture safety studies is needed for developing a protocol for prospective safety studies. We hope that the developed survey form will be used in MSN treatment-related clinical research.

## Author contributions

Conceptualization: Jungtae Leem, Sang-Hoon Yoon

Data curation: NA

Formal analysis: NA

Funding acquisition: Jun-Hwan Lee, Jungtae Leem

Investigation: Sang-Hoon Yoon, Jungtae Leem

Methodology: Haebeom Lee, Chan-Young Kwon, Jungtae Leem, Jae-Uk Sul

Project administration: Jungtae Leem, Damin Jeon

Resources: Aesook Shin, Younghee Yun

Software: NA

Supervision: Jun-Hwan Lee, Hyunho Kim, Hee-Geun Jo, Geon-Mok Lee

Validation: Aesook Shin, Younghee Yun

Visualization: NA

Writing – Original draft: Sang-Hoon Yoon, Jungtae Leem, Haebeom Lee, Chan-Young Kwon

Writing – review & editing: Jungtae Leem, Jun-Hwan Lee, Hyunho Kim, Hee-Geun Jo, Haebeom Lee

**Conceptualization:** Sang-Hoon Yoon, Jungtae Leem.

**Funding acquisition:** Jun-Hwan Lee, Jungtae Leem.

**Investigation:** Sang-Hoon Yoon.

**Methodology:** Haebeom Lee, Chan-Young Kwon, Jae-Uk Sul, Jungtae Leem.

**Project administration:** Damin Jeon, Jungtae Leem.

**Supervision:** Hyunho Kim, Hee-Geun Jo, Geon-Mok Lee, Jun-Hwan Lee.

**Validation:** Aesook Shin, Younghee Yun.

**Writing – original draft:** Sang-Hoon Yoon, Haebeom Lee, Chan-Young Kwon, Jungtae Leem.

**Writing – review & editing:** Sang-Hoon Yoon, Haebeom Lee, Chan-Young Kwon, Jungtae Leem.

Jungtae Leem orcid: 0000-0003-3300-5556.

## Supplementary Material

Supplemental Digital Content
