# Supplementary material for: Development of a survey form through Delphi study about adverse events associated with the miniscalpel needle, for application in prospective observational studies regarding safety of miniscalpel needles: Study protocol
Source: Medicine (Baltimore). 2018 Oct 12;97(41):e12736. doi: 10.1097/MD.0000000000012736 (PMC6203508; doi:10.1097/MD.0000000000012736)
Supplement: Supplemental Digital Content [file medi-97-e12736-s001.docx]

**Supplementary 1. Delphi Round 1 Excerption About the kind and definition**

(Close question)

| **각 항목이 ‘도침치료 후 부작용 체크리스트’에 포함되는 것에 대한 의견 설문**  **“What kind of AEs in MSN treatment should be included in the survey form for future MSN safety research”** | | | | |
| --- | --- | --- | --- | --- |
|  | **크게**  **동의하지 않음**  **Strongly disagree**  **1** | **약간**  **동의하지 않음**  **Somewhat disagree**  **2** | **약간**  **동의함**  **Somewhat agree**  **3** | **크게**  **동의함**  **Strongly agree**  **4** |
| **국소이상반응 (Local adverse reaction)** | | | | |
| 1. 국소부위 통증 (pain) | □ | □ | □ | □ |
| 1. 출혈 (hemorrhage) | □ | □ | □ | □ |
| 1. 멍 (bruise) | □ | □ | □ | □ |
| 1. 혈종 (hematoma) | □ | □ | □ | □ |
| 1. 부종 (edema) | □ | □ | □ | □ |
| 1. 수포 (vesicles) | □ | □ | □ | □ |
| 1. 소양감 (pruritis) | □ | □ | □ | □ |
| 1. 발진 (rash) | □ | □ | □ | □ |
| 1. 국소감염 (infection) | □ | □ | □ | □ |
| 1. 신경손상* (nerve damage) | □ | □ | □ | □ |
| 1. 감각이상 (dysesthesia) | □ | □ | □ | □ |
| 1. 운동장애 (movement impairment) | □ | □ | □ | □ |
| 1. 치료부위불편감 (discomfort) | □ | □ | □ | □ |
| 1. 기흉 (pneumothorax) | □ | □ | □ | □ |
| **전신이상반응 (Systemic adverse reaction)** | | | | |
| 1. 증상악화 (disease aggravation) | □ | □ | □ | □ |
| 1. 피로 (needle fatigue) | □ | □ | □ | □ |
| 1. 졸림 (sleepiness) | □ | □ | □ | □ |
| 1. 오심 (procedural nausea) | □ | □ | □ | □ |
| 1. 구토 (procedural vomiting) | □ | □ | □ | □ |
| 1. 두통 (procedural headache) | □ | □ | □ | □ |
| 1. 현훈 (procedural dizziness) | □ | □ | □ | □ |
| 1. 발한 (sweating) | □ | □ | □ | □ |
| 1. 쇼크  (procedural shock) | □ | □ | □ | □ |
| 1. 실신 (syncope) | □ | □ | □ | □ |
| 1. 호흡곤란 (dyspnea) | □ | □ | □ | □ |
| 1. 부정맥 (arrythmia) | □ | □ | □ | □ |
| 1. 전신의 통증 (procedural pain) | □ | □ | □ | □ |
| 1. 수면장애 (sleep disorder) | □ | □ | □ | □ |
| 1. 불안 (procedural anxiety) | □ | □ | □ | □ |
| 1. 경련 (convulsion) | □ | □ | □ | □ |
| 1. 전신감염 (post procedural infection) | □ | □ | □ | □ |
| 추가의견  Additional opinion | 추가해야 하는 항목이 있거나 기타 의견이 있으면 **해당항목의 정의와 함께** 여기에 입력해 주세요.  In addition to hemorrhage and pain, what other AEs need to be defined by consensus? (Write with the proper definition) | | | |

| **각 부작용 항목에 대해 ‘별도의 보고 기준’이 필요하다고 생각하는 정도에 따라**  **해당 숫자 밑의 □를 ■로 변경해 주십시오**  **“Do you think we should reach an additional agreement on the definition of AEs?"** | | | | |
| --- | --- | --- | --- | --- |
|  | **크게**  **동의하지 않음**  **Strongly disagree**  **1** | **약간**  **동의하지 않음**  **Somewhat disagree**  **2** | **약간**  **동의함**  **Somewhat agree**  **3** | **크게**  **동의함**  **Strongly agree**  **4** |
| **국소이상반응 (Local adverse reaction)** | | | | |
| 1. 국소부위 통증 (pain) | Discuss on open question | | | |
| 1. 출혈 (hemorrhage) | Discuss on open question | | | |
| 1. 멍 (bruise) | □ | □ | □ | □ |
| 1. 혈종 (hematoma) | □ | □ | □ | □ |
| 1. 부종 (edema) | □ | □ | □ | □ |
| 1. 수포 (vesicles) | □ | □ | □ | □ |
| 1. 소양감 (pruritis) | □ | □ | □ | □ |
| 1. 발진 (rash) | □ | □ | □ | □ |
| 1. 국소감염 (infection) | □ | □ | □ | □ |
| 1. 신경손상* (nerve damage) | □ | □ | □ | □ |
| 1. 감각이상 (dysesthesia) | □ | □ | □ | □ |
| 1. 운동장애 (movement impairment) | □ | □ | □ | □ |
| 1. 치료부위불편감 (discomfort) | □ | □ | □ | □ |
| 1. 기흉 (pneumothorax) | □ | □ | □ | □ |
| **전신이상반응 (Systemic adverse reaction)** | | | | |
| 1. 증상악화 (disease aggravation) | □ | □ | □ | □ |
| 1. 피로 (needle fatigue) | □ | □ | □ | □ |
| 1. 졸림 (sleepiness) | □ | □ | □ | □ |
| 1. 오심 (procedural nausea) | □ | □ | □ | □ |
| 1. 구토 (procedural vomiting) | □ | □ | □ | □ |
| 1. 두통 (procedural headache) | □ | □ | □ | □ |
| 1. 현훈 (procedural dizziness) | □ | □ | □ | □ |
| 1. 발한 (sweating) | □ | □ | □ | □ |
| 1. 쇼크  (procedural shock) | □ | □ | □ | □ |
| 1. 실신 (syncope) | □ | □ | □ | □ |
| 1. 호흡곤란 (dyspnea) | □ | □ | □ | □ |
| 1. 부정맥 (arrythmia) | □ | □ | □ | □ |
| 1. 전신의 통증 (procedural pain) | □ | □ | □ | □ |
| 1. 수면장애 (sleep disorder) | □ | □ | □ | □ |
| 1. 불안 (procedural anxiety) | □ | □ | □ | □ |
| 1. 경련 (convulsion) | □ | □ | □ | □ |
| 1. 전신감염 (post procedural infection) | □ | □ | □ | □ |
| 추가의견  Additional opinion | 추가해야 하는 항목이 있거나 기타 의견이 있으면 **해당항목의 정의와 함께** 여기에 입력해 주세요.  In addition to hemorrhage and pain, what other AEs need to be defined by consensus? (Write with the proper definition) | | | |

(Open Question)

| 1. (개방형) 본인이 생각하는 도침으로 인한 부작용으로써의 출혈의 보고기준  What do you think should be the appropriate definition of hemorrhage in the context of safety research? |
| --- |
| 여기에 기입해 주세요 (Please write it here) |
| 1. (개방형) 출혈을 정의하기 위해 강도, 시간 외에 추가적인 변량이 필요하다면 여기에 기입해 주세요  What should be included in the criteria when defining bleeding? |
| 여기에 기입해 주세요 (Please write it here) |
| 1. (개방형) 본인이 생각하는 도침으로 인한 부작용으로써의 통증의 보고기준  What do you think should be the appropriate definition of pain in the context of safety research? |
| 여기에 기입해 주세요 (Please write it here) |
| 1. (개방형) 통증을 정의하기 위해 강도, 시간 외에 추가적인 변량이 필요하다면 여기에 기입해 주세요  What should be included in the criteria when defining pain? |
| 여기에 기입해 주세요 (Please write it here) |
